# Supplementary material for: Multi-Omics Analysis Reveals the Gut Microbiota Characteristics of Diarrheal Piglets Treated with Gentamicin
Source: Antibiotics (Basel). 2023 Aug 22;12(9):1349. doi: 10.3390/antibiotics12091349 (PMC10525804; doi:10.3390/antibiotics12091349)
Supplement: Supplementary file 1 [file antibiotics-12-01349-s001.zip › antibiotics-2512553-supplementary.pdf]

Supplemental Table S1. Primers used in the study

Primer	Nucleotide sequence (5′-3′)	Product Size (bp)
MUC4		
Forward	GTGCCTTGGGTGAGAGGTTA	367
Reverse	CACTCTGCCGTTCTCTTTCC	
Total bacteria		
Forward	ACTCCTACGGGAGGCAGCAG	200
Reverse	ATTACCGCGGCTGCTGG	
Escherichia coli		
Forward	CATGCCGCGTGTATGAAGAA	96
Reverse	CGGGTAACGTCAATGAGCAAA	
Probe	AGGTATTAACTTTACTCCCTTCCTC	

Supplemental Table S2. Ingredient composition of the experimental diets (%, as–fed basis)

Ingredient	Diet
Corn	52.42
Extruded corn	10.00
Soybean meal	13.11
Extruded full-fat soybean	11.00
Whey powder	5.00
Fish meal	4.00
Soybean oil	1.60
Dicalcium phosphate	0.63
Limestone	0.59
Salt	0.30
Permix [1]	0.50
Lys	0.36
Met	0.14
Thr	0.10
Trp	0.05
Choline chloride	0.20
Calculated composition	
Net energy, kcal/kg	2618
Crude protein	17.71
Ca	0.65
P	0.57
SID Lys, %	1.35
SID Met+Cys, %	0.78
SID Thr, %	0.80
SID Trp, %	0.25

Supplemental Table S3. Scoring system for histological changes in the jejunum

score	inflammatory	epithelium	lesion	Lesion degree
0	none	none	none	none
1	lamina propria inflammatory mild infiltration	isolated focal epithelial damage	Mucosal layer	10-25%
2	mild infiltration and multifocal edema	Local injury and epithelial cytopenia	Mucosa and submucosa	26-50%
3	Extensive inflammatory infiltration, glandular separation and edema	extensive damage deep into the bowel wall	transmural	> 50%

Supplemental Table S4. Effects of gentamicin on growth performance of ETEC–challenged pigs

Item	NC	ETEC	ETEC+Gen	SEM	*P*-value
Initial BW, kg	10.85	10.93	10.86	0.25	0.99
D 0 BW, after acclimatization, kg	11.92	11.74	11.89	0.25	0.82
D 5 BW, before euthanized, kg	14.58[a]	13.42[b]	13.93[ab]	0.28	0.03
Weight gain, kg	2.67[a]	1.67[b]	2.04[b]	0.10	＜0.01
ADG, g	532[a]	334[b]	406[b]	23	＜0.01
ADFI, g	737	667	726	22	0.06
G:F	0.73[a]	0.51[b]	0.57[b]	0.03	＜0.01

Supplemental Table S5. Significant taxonomic variation at the phylum level

Item	NC	ETEC	ETEC+Gen	SEM	*P*-value
Tenericutes	0.067648	0.021658	0.01276	0.017	0.007
WPS-2	0.000093	0.000028	0.000226	0.00005	0.014
Rokubacteria	0.000246	0.000056	0.001047	0.00030	0.021
Euryarchaeota	0.000111	0.000072	0.000529	0.00014	0.038
Nitrospirae	0.000336	0.000083	0.001327	0.00037	0.047

Supplemental Table S6. Significant taxonomic (top 20) variation at the genus level

Item	NC	ETEC	ETEC+Gen	SEM	*P*-value
uncultured_bacterium_f_bacteriap25	0.000025	0.000002	0.000107	0.000032	0.002
[Eubacterium]_brachy_group	0.000038	0.000176	0.000073	0.000041	0.006
Haliangium	0.000028	0.000002	0.000137	0.000041	0.007
Ruminiclostridium	0.006308	0.008881	0.004505	0.001270	0.009
Enhydrobacter	0.000044	0	0.000094	0.000027	0.009
Mycoplasma	0.052477	0.005341	0.000301	0.016616	0.010
Chryseobacterium	0.00001	0.000016	0.000134	0.000040	0.010
uncultured_bacterium_o_Mollicutes_RF39	0	0	0.000024	0.000008	0.010
uncultured_bacterium_o_Microtrichales	0.000015	0	0.000126	0.000040	0.011
Breznakia	0	0.000002	0.000021	0.000007	0.012
Variovorax	0.000052	0.000016	0.000184	0.000051	0.012
uncultured_bacterium_f_Intrasporangiaceae	0.000076	0.00002	0.000178	0.000046	0.014
uncultured_bacterium_p_WPS-2	0.000093	0.000028	0.000226	0.000058	0.014
Candidatus_Solibacter	0.000136	0.000038	0.000608	0.000176	0.017
Tepidimicrobium	0.003124	0.004565	0.002276	0.000668	0.017
uncultured_bacterium_f_Rikenellaceae	0.000058	0.000012	0.000391	0.000119	0.017
Hafnia-Obesumbacterium	0.000019	0.000002	0.000114	0.000035	0.019
Ochrobactrum	0.001493	0.001581	0.002281	0.000249	0.019
Butyricimonas	0.000021	0.000002	0.00014	0.000043	0.019

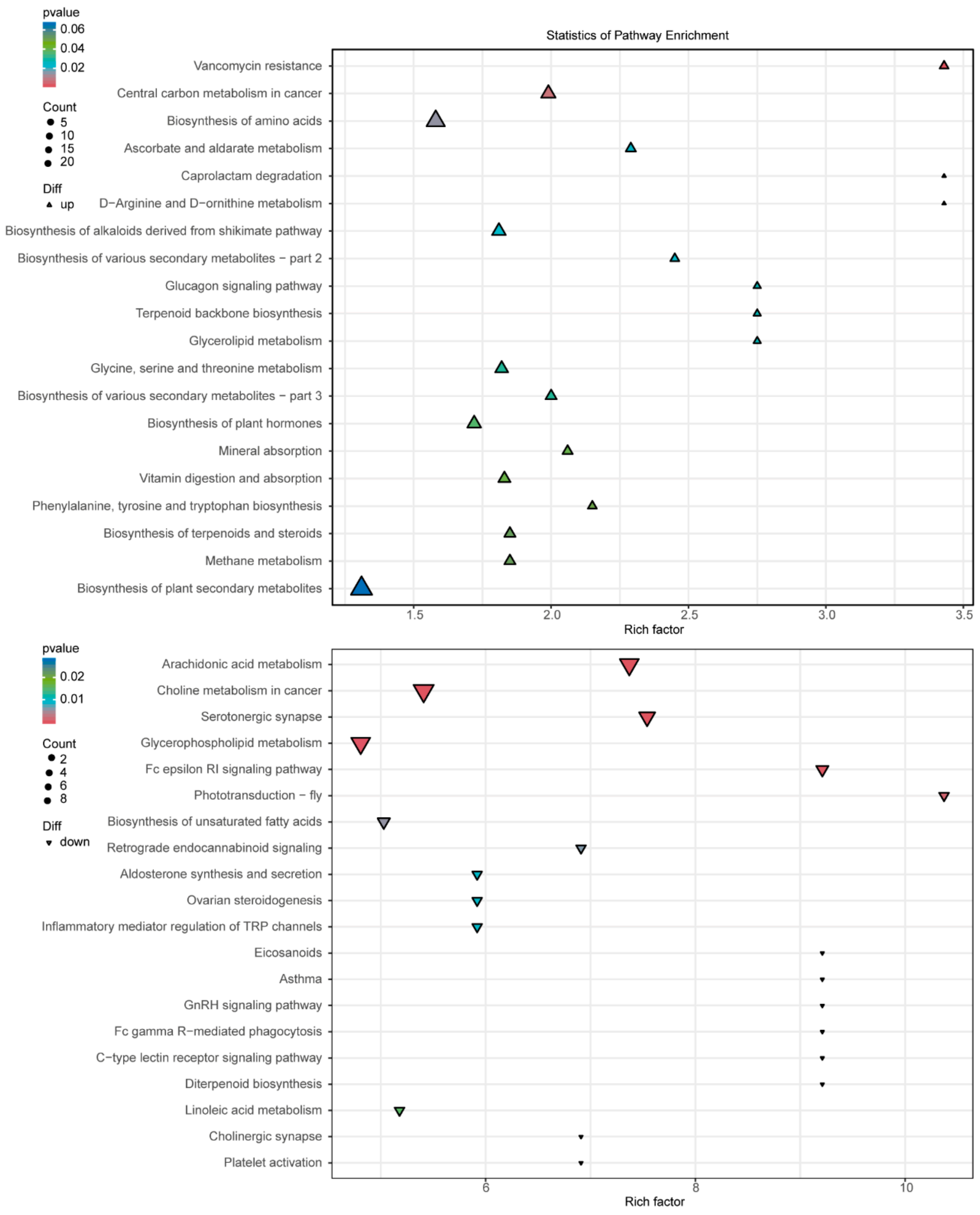

Supplemental Figure S1. The top 20 KEGG enrichment pathway analysis of differentially expressed metabolites between ETEC–challenged and gentamicin-treated piglets.
